# Spatial Mapping of Valence Excited-State Landscapes Using Time-Resolved Shake-Down Spectroscopy

**DOI:** 10.1021/acs.jpca.6c02224

**Published:** 2026-05-22

**Authors:** Henry J. Thompson, Michele Devetta, Davide Faccialà, Rebecca A. Ingle, Stephen T. Pratt, Weronika O. Razmus, Caterina Vozzi, Felix Allum, Michael N. R. Ashfold, Sonia Coriani, Raimund Feifel, Ruaridh Forbes, David M. P. Holland, Daniel Rolles, Richard J. Squibb, Matteo Bonanomi, Carlo Callegari, Marcello Coreno, Miltcho Danailov, Alexander Demidovich, Michele Di Fraia, Cesare Grazioli, Michele Manfredda, Oksana Plekan, Russell S. Minns

**Affiliations:** † School of Chemistry and Chemical Engineering, 7423 University of Southampton, Highfield, Southampton SO17 1BJ, United Kingdom; ‡ CNR-Istituto di Fotonica e Nanotecnologie (IFN), 20133 Milano, Italy; § Department of Chemistry, University College London, London WC1H 0AJ, United Kingdom; ∥ Chemical Sciences and Engineering Division, 1291 Argonne National Laboratory, Lemont, Illinois 60439, United States; ⊥ Deutsches Elektronen-Synchrotron DESY, Notkestraße85, 22607 Hamburg, Germany; # Stanford PULSE Institute, SLAC National Accelerator Laboratory, 2575 Sand Hill Road, Menlo Park, California 94025, United States; ∇ Linac Coherent Light Source, SLAC National Accelerator Laboratory, 2575 Sand Hill Road, Menlo Park, California 94025, United States; ○ School of Chemistry, 1980 University of Bristol, Bristol BS8 1TS, United Kingdom; ◆ Department of Chemistry, Technical University of Denmark, Kgs. Lyngby DK-2800, Denmark; ¶ Department of Physics, 3570 University of Gothenburg, Gothenburg 41296, Sweden; & Department of Chemistry, University of California, Davis, California 95616, United States; ● Science and Technology Facilities Council (STFC), Daresbury Laboratory, Warrington WA4 4AD, United Kingdom; ◊ J.R. Macdonald Laboratory, Department of Physics, 5308 Kansas State University, Manhattan, Kansas 66506, United States; ▲ Elettra-Sincrotrone Trieste S.C.p.A., Basovizza, 34149 Trieste, Italy; □ CNR-ISM, Istituto di Struttura della Materia, Trieste Branch, s.s. 14—km 163,5, in Basovizza Area Science Park, 34149 Trieste, Italy; ^ CNR-Istituto Officina dei Materiali (IOM), Basovizza, 34149 Trieste, Italy

## Abstract

Time-resolved X-ray photoelectron spectroscopy (XPS) is used to track the photodissociation dynamics of 2-iodothiophene following 262 nm excitation. The transient XPS features include both direct ionization of the initially populated excited states and pronounced satellite peaks arising from shake-down processes. While the direct ionization signals exhibit only minimal energy shifts during C–I bond cleavage, the shake-down transitions undergo a substantial, 5 eV, shift over the reaction coordinate. By correlating these shifts with simulated C–I bond lengths, a direct structural mapping is established that reveals the exceptional sensitivity of shake-down channels to molecular geometry. These results demonstrate that shake-down transitions provide a new and powerful probe of ultrafast structural dynamics.

## Graphical abstract

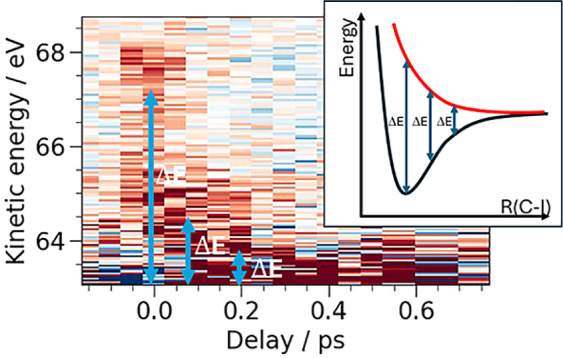

## Introduction

The development of free electron laser (FEL) technologies has significantly enhanced the power of femtosecond resolution X-ray spectroscopies for improving our understanding of photochemical processes. The advantage of X-ray spectroscopies lies in the ability to selectively probe particular atomic sites within a molecule and to monitor subtle changes in the local electronic environment.^1–9^ Recent X-ray photoelectron spectroscopy (XPS) studies have shown that with a sufficiently high signal-to-noise ratio, satellite processes, where there is a commensurate change in the valence electronic structure, such as shake-down transitions, can be observed in time-resolved measurements.^4^ The observation of such satellite processes combines the site selectivity of X-ray spectroscopies with an enhanced sensitivity to changes in the valence electronic structure and has great potential as a probe of photochemical processes. The development of such experimental techniques requires large scale experimental and complementary computational efforts to understand the sensitivity of shake-down to characteristic chemical processes such as bond-breaking reactions.

While early in its development, several characteristic advantages of measuring shake-down transitions are already apparent and further developed in the work presented in this paper. Namely, large energy shifts of several electronvolts are seen as a function of changes in electronic and geometric structure. Allied to this are the site selectivity of the X-ray ionization process and the spin-preserving nature of the shake-down transitions that allow the character of the electronically excited states involved to be identified. It should also be noted that alongside the shake-down peaks, the direct X-ray photoelectron spectrum is also collected at the same time, thereby providing complementary information. All of these advantages and characteristics suggest that shake-down spectroscopy will increasingly become recognized as an exciting new opportunity within the time-resolved spectroscopy toolbox.

Satellite transitions in XPS measurements, such as shake-down from core^10,11^ or valence^12^ excited states, and shake-up from electronic ground states,^13–15^ derive from the interaction of an outgoing core-ionized electron with the outer bonding/valence electrons. Within the sudden approximation, the monopole created by the core electron escaping the molecular potential drives a valence transition. Shake-up leads to the formation of a core-ionized state in which one of the valence electrons has also been excited. This reduces the kinetic energy of the ejected electron with respect to that of the primary photoelectron transition. Shake-down is the reverse process, where a valence electron makes a transition to a more tightly bound orbital upon core ionization. Shake-down can only occur when ionizing electronically excited states and leads to an increase in the kinetic energy of the outgoing electron.^4,12^ Shake-down transitions therefore provide a novel route to monitor time-dependent changes in the character of the valence excited states during chemical reactions, and their presence results in the appearance of additional peaks in the photoelectron spectrum at electron kinetic energies higher than expected from the primary XPS transition(s). This higher kinetic energy leads to the shake-down features generally being free from spectral congestion to which TR-XPS and Auger–Meitner measurements are often prone.^2,3,5,6,9,16^

As the experiments described herein focus on valence excited states, we will limit the rest of our discussion of shake-down processes to these cases and ignore excited states produced via core-to-valence excitations. To illustrate the relationship between the primary XPS photoelectron peak and those due to shake-down processes, a static representation of the two processes is presented in Figure 1. Ionization of the electronic ground state by an X-ray photon gives rise to a photoline with kinetic energy eKE_GS_, related to the formation of a cation with a core hole. Population of a valence excited state at an energy Δ*E* above the ground state (GS), for example, by the absorption of a UV photon, creates a valence hole in the neutral molecule together with an electron in a previously empty valence orbital. Direct ionization of the valence excited state (ES) by the absorption of an X-ray photon of the same energy may lead to the release of a core electron with kinetic energy eKE_ES_ and the formation of a cation with a core hole and a valence hole. The relative energies of eKE_ES_ and eKE_GS_ depend on the character of the ground and excited states and how excitation changes the local electronic environment of the atom being ionized.^3^ If X-ray ionization from the excited state results in shake-down (SD), the valence hole is filled, and the final cation state has the same single core-hole character as that populated following ionization of the electronic ground state. The electron kinetic energy from the shake-down process, eKE_SD_, is increased relative to eKE_GS_ by the valence excitation energy, Δ*E*, creating a distinct peak in the spectrum that is, at least initially, well separated from the primary XPS signal.

**Figure 1:**
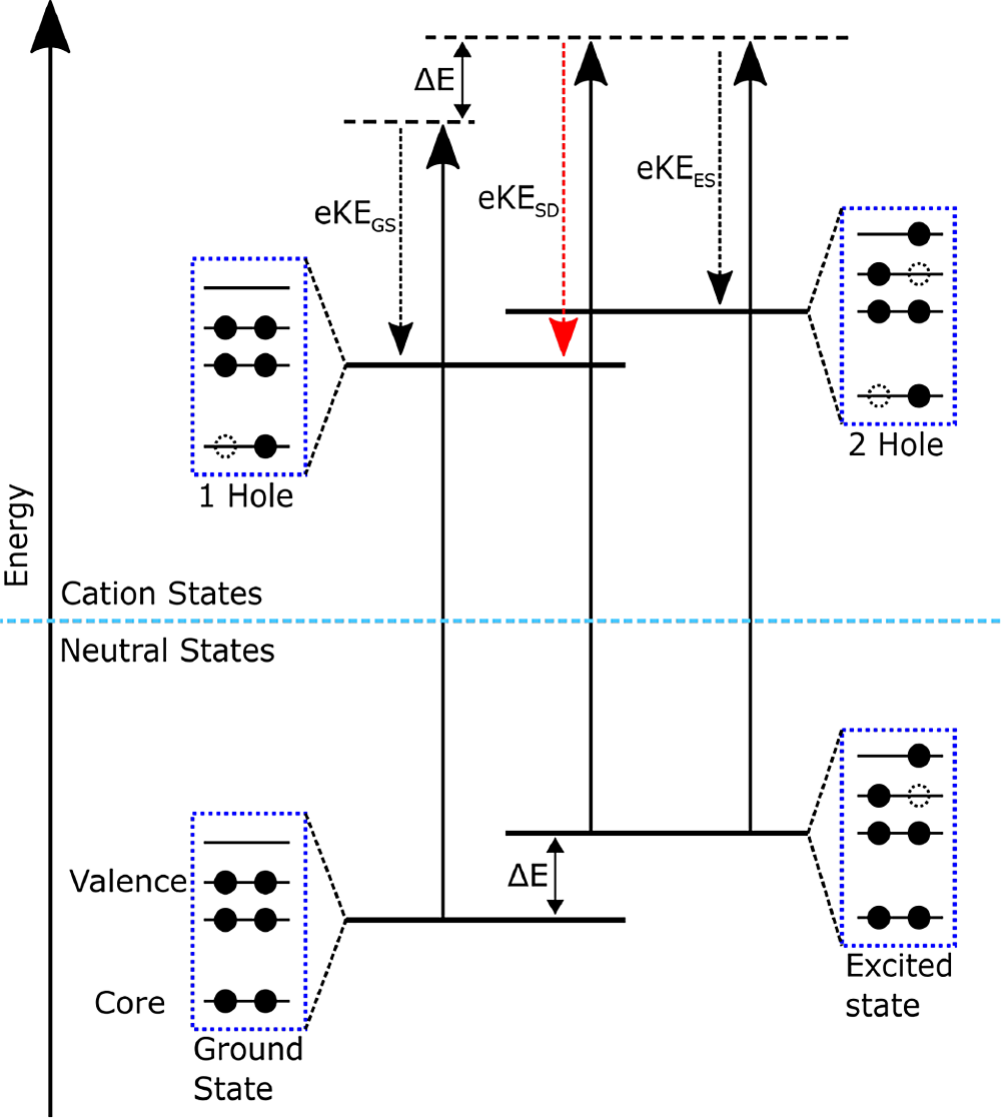
Energy level diagram of X-ray-driven core electron ionization events available in a TR-XPS experiment. For each energy level, a cartoon electron configuration is expanded at the side to highlight the formation of core and/or valence holes, represented by unfilled dashed circles, following ionization by an X-ray photon (black solid arrows) or through valence excitation. Ionization of the electronic ground state by an X-ray photon gives rise to a photoelectron with kinetic energy eKE_GS_. Population of a valence excited state at an energy Δ*E* above the ground state creates a valence hole. Ionization of the valence excited state by an X-ray photon of the same energy leads to the release of an electron with kinetic energy eKE_ES_. If X-ray ionization results in shake-down, the valence hole is filled during the ionization process, and the kinetic energy of the outgoing electron, eKE_SD_ (red arrow), is increased relative to eKE_GS_ by the valence excitation energy, Δ*E*.

In a time-resolved (TR) experiment where changes in geometry and/or electronic state lead to changes in Δ*E*, the positions of the shake-down peaks can provide a sensitive probe of valence excited state character, as recently reported in a combined experimental and theoretical study of the excited state dynamics of CS_2_.^4^ Measurements and accompanying theoretical modeling of shake-down transitions in CS_2_, following ionization from the S 2p orbitals, have shown how changes in the character of the valence excited states, following internal conversion processes, result in separate shake-down features in the XPS spectrum. Detailed analysis of the spectra, through comparison to ab initio models, enabled specific electronic states involved in the excited state dynamics to be identified and allowed derivation of propensity rules for the spin multiplicity of the states involved in the transitions.^4^

To investigate the sensitivity of the shake-down transitions to bond dissociation reactions, TR-XPS experiments of UV (262 nm) excited 2-iodothiophene (2-IT, C_4_H_3_SI) have been performed. Excitation at 262 nm leads to the following reaction scheme:
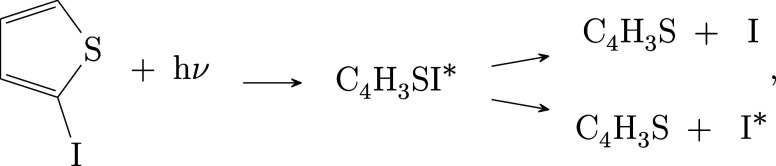
where I and I* represent the ground and spin–orbit excited states of atomic iodine formed in conjunction with the electronic ground state of the thiophenyl radical (C_4_H_3_S).^17^

The character of the initially excited state is subject to some disagreement in the literature, with multiple states contributing to the overall absorption spectrum. The UV absorption spectrum of 2-IT is dominated by a strong, thiophene ring-localized, ππ* absorption band with a peak maximum around 240 nm that extends to approximately 260 nm.^18^ Overlapping with the ππ* band, and extending to longer wavelengths, there is a weaker band associated with excitation of C–I bond-localized (n/π)­σ* states that share strong similarities with many alkyl halides.^19–21^ Recent experiments have disagreed about the character of the initially excited state at a pump wavelength of 262 nm, with Toulson et al.^22^ suggesting that excitation is dominated by ππ* contributions, while those of Razmus et al.^18^ indicate that the (n/π)­σ* states dominate with only a 4% contribution from the ππ* state. These assignments were based on measurements of product states and on theoretical models that ignored the effects of spin–orbit coupling on the transition dipole moments and should therefore be considered tentative. To test which state dominates the initial excitation, we exploit the site selectivity of XPS measurements to changes in local environment and record spectra when exciting the (n/π)­σ* transition in 2-IT at 262 nm. TR-XPS measurements of 2-IT have been carried out at both the iodine 4d and sulfur 2p edges. No time-dependent changes were observed in the sulfur 2p spectrum, beyond a multiphoton ionization-induced depletion of the ground-state signal at time zero (see Supporting Information (SI)). The lack of any new peaks in the spectrum indicates that there is little change in the local environment of the sulfur atom upon UV excitation. Any contribution of the ππ* states to the overall excited state population is therefore minimal, consistent with the previous estimates of Razmus et al.^18^ Significant changes were observed in the iodine 4d spectrum, which is the focus for the remainder of the manuscript, and confirm the dominant (n/π)­σ* character of the initial transition in 2-IT at 262 nm.

## Methods

Experiments were performed using the high-resolution magnetic bottle spectrometer at the Low Density Matter (LDM) beamline at the FERMI Free Electron Laser in Trieste (Italy).^23–26^ An X-ray energy of 120 eV (10.33 nm) was used to ionize the iodine 4d electrons with a resultant electron kinetic energy around 60 eV. The probe energy was chosen to be close to the maximum ionization cross-section for the I­(4d) shell to provide a high-contrast signal. A retardation voltage of 47 V was applied to reduce the electron kinetic energy at the detector and hence improve the spectral resolution. Spectra were recorded with the FEL running at 50 Hz and the UV (262 nm, 4.73 eV) pump laser running at 25 Hz, thereby providing sequential pump-on and pump-off measurements. At each time delay, *t*, a difference spectrum, Δ*S*(*t*), was obtained by subtracting the pump-off spectrum from the pump-on spectrum. See SI for full experimental details.

## Results and Discussion

An overview of the experimental differential signal obtained from the TR-XPS data from 2-IT following 4.73 eV (262 nm) excitation and ionization of the I­(4d) level with a 120 eV (10.33 nm) probe is presented in Figure 2. The differential (pump-on minus pump-off) color map (a) allows the transient photoelectron features to be examined as a function of electron kinetic energy and pump–probe delay. Averaged differential spectra at long (c) and short (b) delays show the spectral features present at various delays and highlight key differences in the peaks observed. The energy range is split into two regions that show the primary core electron photolines at kinetic energies below 63.7 eV and the shake-down signals at kinetic energies above 63.7 eV. The intensity scale is multiplied by 10 for kinetic energies above 63.7 eV to improve visibility of the shake-down signals and allow the same color scale to be used across the full figure.

**Figure 2:**
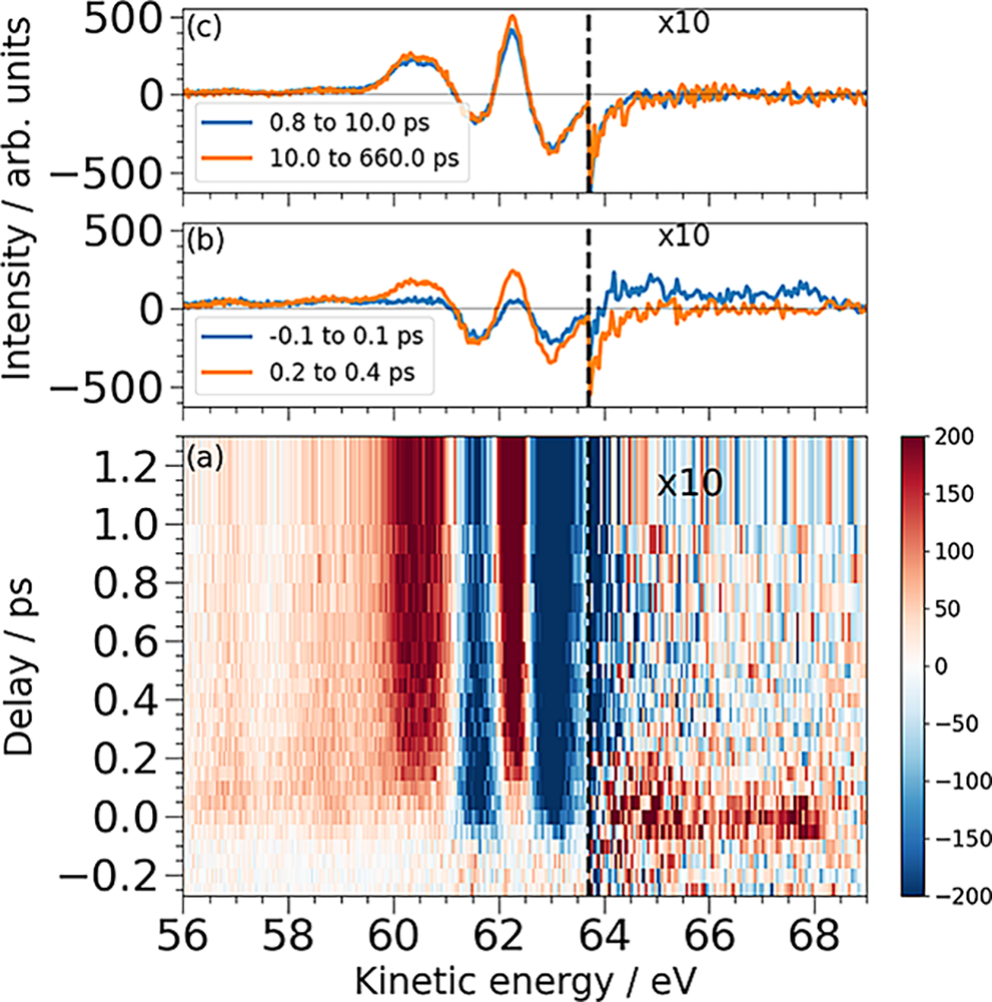
Differential TR-XPS measurements, Δ*S*, of 2-iodothiophene following 262 nm excitation and ionization with a 120 eV probe. Spectra obtained at (c) long and (b) short delays provide a breakdown of the spectral features at various different delay ranges. (a) Provides the full time-dependent data as a colormap. For kinetic energies above 63.7 eV, the intensity scale has been multiplied by 10.

In Figure 2a, the depletion of the ground state of 2-IT by the pump photon is seen as the negative intensity (blue) features in the spectrum centered at 61.5 and 63.3 eV. These kinetic energies are consistent with the known binding energies of the I 4d_5/2_ and I 4d_3/2_ levels of 2-IT at 56.78 and 58.43 eV, respectively.^27^ The depletion of the ground-state signal coincides with the appearance of transient features at lower and higher electron kinetic energies. The signal seen in the region between 56 and 59.5 eV appears at time zero and is assigned to FEL ionization of 2-IT cation states that are populated through multiphoton ionization with the UV pump. This signal is seen to decay over a picosecond time scale due to dissociative ionization processes, as will be discussed later in the manuscript. We note here that while this indicates that there are multiphoton processes occurring, the spectral separation of these signals means we can isolate them from the neutral dynamics of interest. The large shift seen for the ion states populated by multiphoton ionization is in the opposite direction to that of shake-down features associated with the neutral excited states of most importance here. This presents another key advantage of this technique over many other structural probes, for example, X-ray/electron scattering or Coulomb explosion imaging, where multiphoton excitation effects would lead to overlapping signals in the data that often cannot be isolated.

The region between 59.5 and 62.6 eV contains two peaks of much higher intensity and shows transient changes on the fs time scale. Positive signals in this region are assigned to the transient excitation of the neutral excited states of 2-IT and the formation of atomic iodine as a final product of the photolysis reaction, with previous measurements indicating that ground state iodine is the dominant product, accounting for 77% of the overall yield.^22^ Previous studies using synchrotron radiation indicate that the electron kinetic energies of the 4d orbitals in atomic iodine are expected to be observed between 59.5 and 63.3 eV when ionized with a 120 eV probe,^28,29^ matching the peak positions in the long delay spectrum plotted in Figure 2c. As the thiophenyl radical cofragment does not contain an iodine atom, the present measurement will be blind to this component once formed.

Over the first 200 fs, weak pump–probe signals are observed in the kinetic energy range between 63.7 and 68.2 eV. These kinetic energies are greater than can be expected through direct core ionization of the valence excited state by the X-ray photon alone and are therefore assigned to shake-down processes in which core ionization is accompanied by a valence electron transition to fill the valence hole. The shake-down signals occur over a continuum of energies that are initially observed around 68 eV (approximately 4.7 eV above the ground-state XPS peak energy and equal to the UV pump-photon energy used in the experiment) and subsequently move to lower kinetic energies. While the initial energy shift of 4.7 eV could be explained through the formation of sidebands when the pump and probe pulses overlap in time, such a process would produce replicas of the ground state spectrum shifted by the photon energy to both higher and lower kinetic energies. The lack of structure in the spectrum and the lack of any features shifted by the photon energy to lower kinetic energies suggests that any such contribution is small. Sideband formation could also not explain the broad continuum observed on the high kinetic energy side of the electronic ground state such that any sideband contribution would not impact the analysis presented here. After approximately 200 fs, the energy of the shake-down signal has reached the point that is associated with the ground state depletion where it becomes obscured. Overlap of the XPS photolines and shake-down peaks at later delays with the ground state depletion signals makes analysis challenging and requires routes to extraction of data that can remove the ground state contribution effectively.

In order to remove the ground state depletion from the spectrum and extract further details of the transient changes, a time-dependent subtraction of the ground-state contribution was performed. The subtraction was based on measurements of the instrument response function, which defines the rate of the depletion of the ground-state signals, and of the asymptotic product spectrum, which defines the level of ground state depletion in the excitation step.

The photolysis reaction leads to the formation of the thiophenyl radical and atomic iodine on an ultrafast time scale. By measuring the asymptotic difference spectrum at long delays where the reaction is effectively complete (50–650 ps here), it is possible to obtain a spectrum composed of two components, namely ground state 2-IT, *S*_GS_, and atomic iodine, *S*_I_. It should be noted that *S*_GS_ is experimentally observed every time a probe-only spectrum is recorded such that this is a known function and is plotted in Figure 3a. The difference signal obtained at asymptotically long times, Δ*S*(*t* = ∞), is therefore

$$\begin{array}{ll} \Delta S(t=\infty ) & =(1-A)\cdot S_{\mathrm{GS}}+S_{I}-S_{\mathrm{GS}}\\ & =S_{I}-A\cdot S_{\mathrm{GS}} \end{array}$$ 1

where *A* defines the fraction of molecules that were excited by the pump. Typically, the value of *A* is unknown but controls the size of the negative signal and can be extracted from the asymptotic data. Adapting eq 1 to be a scaled subtraction, Δ*S*_scaled_(*t* = ∞), of *S*_GS_ at asymptotically long pump–probe delays we obtain

$$\Delta S_{\mathrm{scaled}}(t=\infty )=(1-A)\cdot S_{\mathrm{GS}}+S_{I}-(1-A_{f})\cdot S_{\mathrm{GS}}$$ 2

where *A*_f_ is the scaling parameter that can be used to obtain the level of ground state depletion apparent in the signal. Utilizing the same approach as that of Thompson et al.^6^ and Warne et al.^30^ on the analysis of Auger and valence photoelectron spectra, *A*_f_ is adjusted to the smallest value that does not generate a meaningful negative signal (depletion) above the spectral noise such that *A*_f_ = *A*. If done appropriately, this process removes all negative features in the asymptotic spectrum and leads to the recovery of the atomic iodine spectrum, i.e.

$$\begin{array}{ll} \Delta S_{\mathrm{scaled}}(t=\infty ) & =(A_{f}-A)\cdot S_{\mathrm{GS}}+S_{I}\\ & =S_{I} \end{array}$$ 3

**Figure 3:**
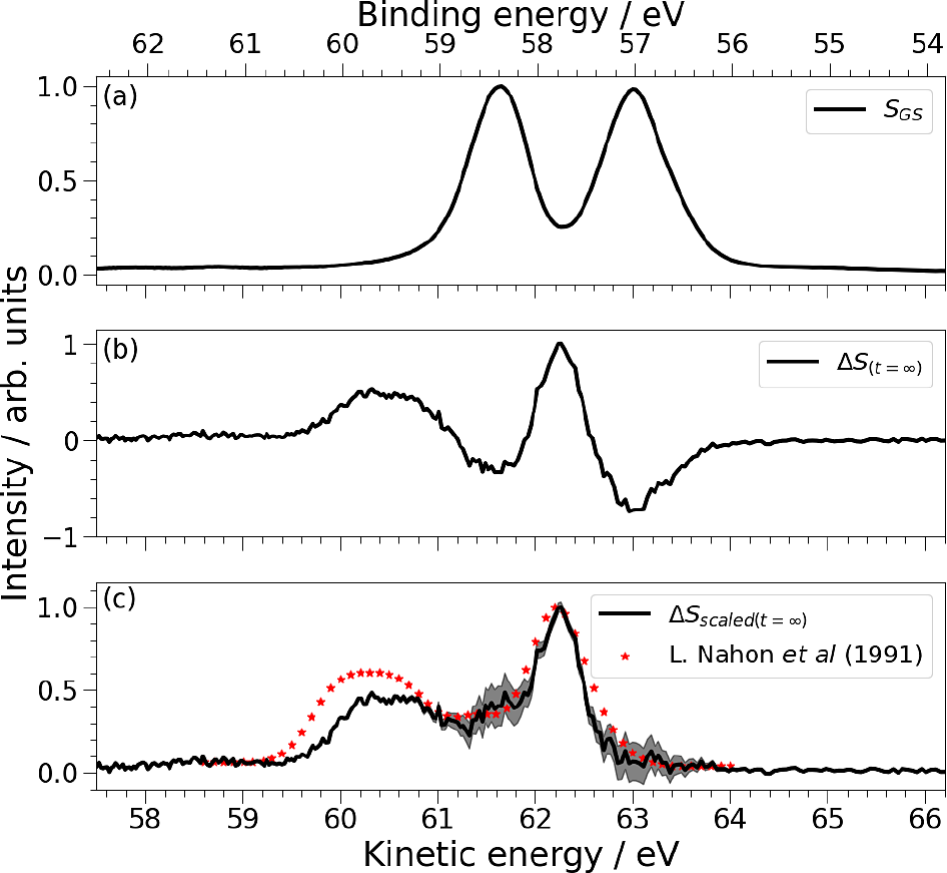
(a) Ground state photoelectron spectrum of 2-IT, *S*_GS_. (b) Difference photoelectron spectrum obtained at asymptotically long pump–probe delays (50–650 ps), Δ*S*(*t* = ∞), consisting of the iodine product and ground state depletion signals. (c) Scaled subtraction, Δ*S*_scaled_(*t* = ∞), obtained with an *A*_f_ value of 0.17 ± 0.03. The shaded region represents the ±0.03 uncertainty of the *A*_f_ value. The red data points show the digitized atomic iodine spectrum obtained at a probe energy of 100 eV, reproduced with permission from Nahon et al.,[Bibr ref28] 1991 American Physical Society. The binding energy reported by Nahon et al.^28^ is plotted at the top of the figure. This binding energy is subtracted from our probe energy to allow comparison on the kinetic energy scale used throughout the rest of the manuscript.

The results of the analysis are presented in Figure 3, where (a) is the ground state spectrum, *S*_GS_, (b) is the difference spectrum at asymptotically long delays, Δ*S*(*t* = ∞), and (c) is the result of the scaled subtraction, Δ*S*_scaled_(*t* = ∞), with a retrieved value of *A*_f_ = 0.17 ± 0.03. The 0.03 uncertainty is represented as the shaded regions of Figure 3c, showing how this has a very limited effect on the shape of the iodine spectrum so obtained.

The scaled subtracted spectrum obtained is in good agreement with the atomic iodine 4d spectrum measured with synchrotron radiation by Nahon et al.,^28^ which is plotted as the red data points in Figure 3c, giving confidence that the process is robust and that atomic iodine is the dominant iodine-containing fragment obtained at long delays, consistent with previous studies of 2-IT using similar pump wavelengths.^18,22,31^ We note that the spectrum of Nahon et al.^28^ is only of the ground spin–orbit state of atomic iodine (I) and not I*, such that we may expect differences between the two. However, given the very similar expected spectrum of I* (based on measurements of the isoelectronic system Xe^+^ ^29^) and that previous measurements by Toulson et al.^22^ indicate that I is the dominant product (accounting for 77% of the fragments produced when photolyzing at 268 nm), these differences are expected to be small.

Alongside the molecular XPS signal, ionization of the helium seed gas used to aid transport of the molecule into the gas phase was also measured. During temporal overlap of the UV and X-ray pulses used in the experiment, sidebands on the helium peak are observed. As sidebands can only be observed during temporal overlap of the pump and probe pulses, the time-dependent intensity can be used to extract the instrument response function (IRF) independent of the molecular signals. Importantly, the helium and 2-IT spectra were obtained within the same measurement, with the peaks due to helium appearing at a higher electron kinetic energy. Fits to the intensity profile of the helium signal are shown in SI and give a Gaussian IRF width of σ = 66 ± 2 fs, and a delay *t*_0_ uncertainty of ±2 fs.

As depletion of the electronic ground state features in the measurements of 2-IT is controlled by the integrated intensity of the pump and probe pulses, the depletion will have a temporal profile that follows the integrated intensity, or cumulative distribution function, of the Gaussian IRF measured in the helium sidebands with a final amplitude of *A*_f_ as defined in the earlier analysis of the asymptotic spectrum. Based on these two parameters, a time-dependent scaled subtraction of the probe-only, ground state, signal from the pump–probe spectrum was performed such that it removes the correct amount of ground state contributions for a given delay and allows for isolation of dynamic signals in an effective and almost background-free manner. Mathematically, the following operation is performed, which is equivalent to a time-dependent background subtraction

$$S\left(t\right)=\Delta S\left(t\right)+A_{\mathrm{cdf}}\left(t\right)\cdot S_{\mathrm{GS}}$$ 4

where *A*_cdf_(*t*) is the cumulative distribution function of the Gaussian IRF with amplitude *A*_f_, Δ*S*(*t*) is the experimental difference spectrum as presented in Figure 2c, and *S*(*t*) is the resultant spectrum that will be free from any ground state background contributions and contain signals only associated with the excited state process and dissociation products. This becomes especially useful to resolve the relatively small shake-down signals that are obscured by the strong depletion of the ground state features.

The results of time-dependent subtraction are plotted in Figure 4a. Panels (c) and (b) show the average spectra obtained over the same long and short delays used in Figure 2. The time-dependent maps and average spectra show much more clearly the growth of the iodine fragment signal and the evolution of the shake-down signal toward lower kinetic energies.

**Figure 4:**
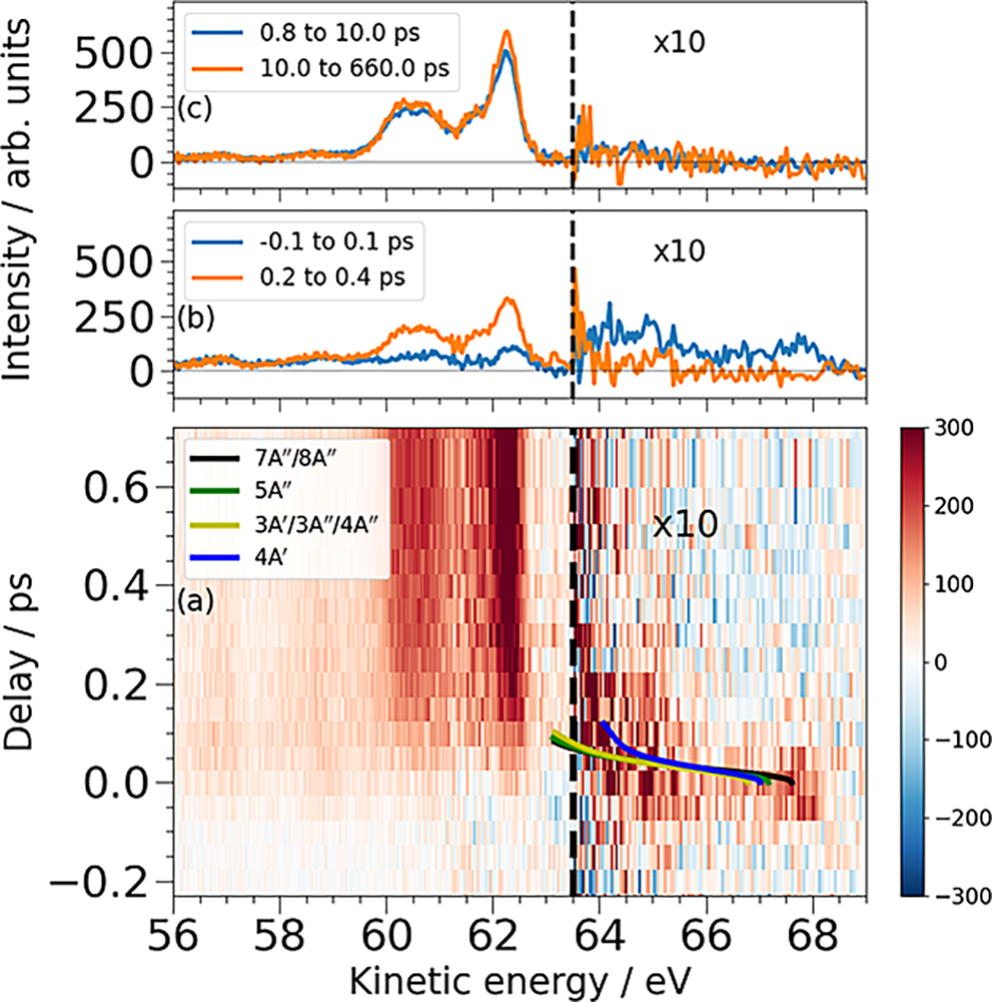
Time-resolved X-ray photoelectron spectrum of 2-IT (262 nm excitation and ionized with a 120 eV probe) following the time-dependent scaled background subtraction described by eq 4. Spectra obtained at (c) long and (b) short delays provide a breakdown of the spectral features at different delay ranges. (a) provides the full time-dependent data as a colormap. The solid lines plotted in panel (a) represent the expected peak positions based on the simulations described in the main text. The color coding of the lines labels the valence excited states and matches the assignment of Figure 6a.

The scaled subtraction has removed the ground state depletion from the iodine product region; thus the full width of the shake-down signal can be integrated to obtain information on product formation time. The temporal profile for the formation is plotted in Figure 5a and shows a two-component rise. In common with the measurements of Razmus et al.,^18^ the intensity of the product signal is fit to a dual logistic function

$$\begin{array}{l} I_{I}=\sum_{n=1}^{2}A_{n}\left[\frac{1}{1+e^{-k_{n}(t-t_{n})}}\right] \end{array}$$ 5

that characterizes an appearance time, *t*_*n*_, as the center of the rise of each component relative to the pump–probe delay time, *t*, as well as an associated exponential rise-time constant, *k*_*n*_. The amplitudes of the two components in the fit, *A*_*n*_, are also obtained. The result of the fit is plotted as solid lines in Figure 5a. The dominant component in the fit is prompt, with an appearance time of 78 ± 7 fs. A secondary delayed component rises over a much longer time scale and appears on a ∼0.67 ps time scale. The amplitudes of the two components indicate that the delayed component contributes approximately 30% of the overall signal at delays greater than 2 ps. Previous studies performed at the same pump wavelength also observed a secondary slow rise in the yield of atomic iodine, which was attributed to a minor contribution following initial excitation into a predissociative ππ* state.^18^ In the previous measurement, the atomic iodine yield increased on a longer time scale than seen in the present work and made a significantly lower relative contribution to the total yield (4%).^18^ These differences in yield and time scale suggest that the origins of the delayed signals observed in the previous studies and here are different.

**Figure 5:**
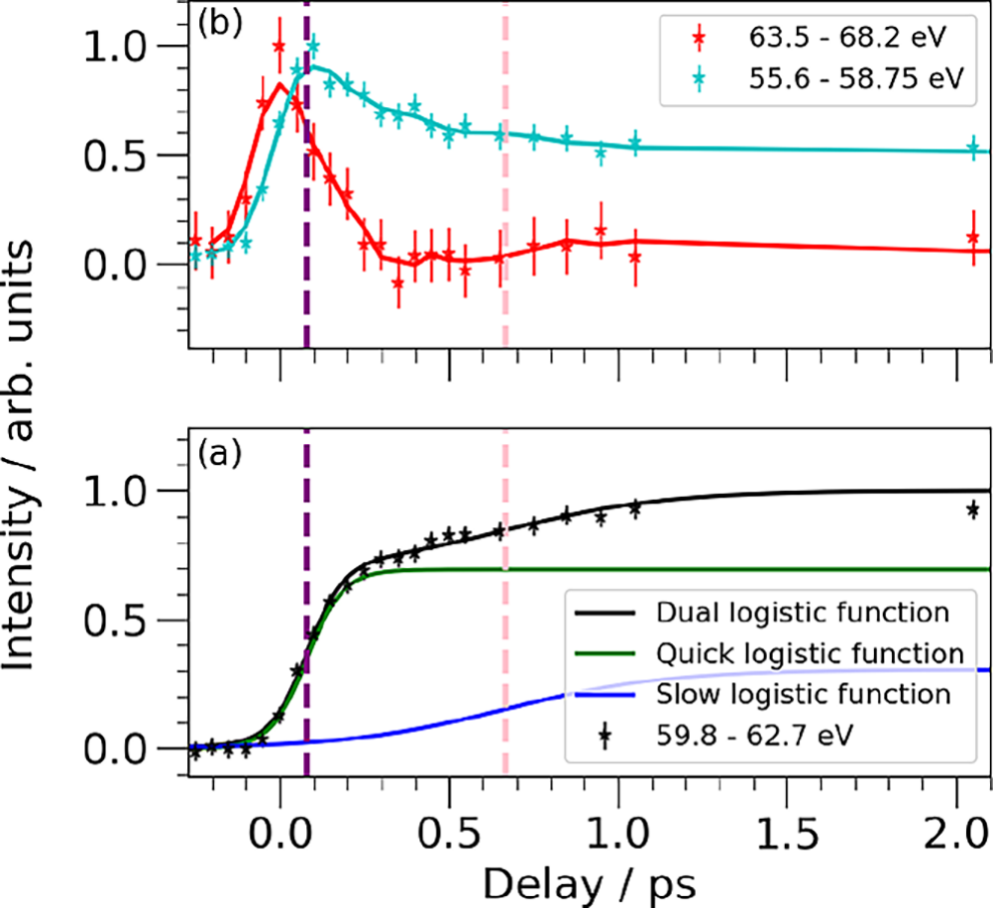
Integrated intensity profiles for (a) atomic iodine and (b) bound molecular signals. The data are plotted with 2σ confidence intervals obtained from a bootstrap analysis. (b) Integrated intensity over the 55.6–58.75 eV kinetic energy range assigned to the 2-IT cations populated via multiphoton UV absorption (light blue), and the integrated intensity of the shake-down signal between 63.5 and 68.2 eV (red). The two signals are normalized to their intensity maximum, and a three-point moving average (solid line) is included as a guide to the eye. (a) Integrated intensity of the iodine product signal observed between 59.8 and 62.7 eV (black). The solid black line represents a fit to eq 5 with the prompt and delayed components plotted as green and dark blue lines, respectively. The appearance times of the prompt and delayed logistic functions derived from the fit are shown as purple and pink dashed vertical lines, respectively.

To determine the origins of the prompt and delayed channels in the present experiment, we also plot the intensity profiles of the neutral excited states (as defined by the shake-down signal observed between 63.5 and 68.2 eV) and the cation states populated by multiphoton absorption of the UV pump (as defined by the signal between 55.6 and 58.75 eV) in Figure 5b. The solid lines are a three-point moving average through the data and are simply presented as a guide to the eye. The decay in the shake-down signal intensity profile aligns well with the prompt formation of atomic iodine signal. We therefore assign this prompt contribution of the atomic iodine signal to neutral dissociation from the initially populated (n/π)­σ* states. The decay in the cation state signal occurs over a longer time scale that matches that seen in the iodine formation. This delayed formation of atomic iodine is therefore assigned to dissociative ionization of cation states populated via multiphoton absorption of the UV pump. Any small (∼4% of the prompt signal based on previous measurements at this wavelength^18^) contribution to the delayed channel from dissociation of initially populated ππ* states of 2-IT will spectrally overlap with the much larger signals described here, making it difficult to identify within the present data set. As the focus of the manuscript is on the shake-down signals associated with the neutral dynamics, the longer-term changes associated with the cation states will not be discussed further.

To understand the spectral position and delay-dependent shifts of the shake-down signals, classical dynamics simulations of the photolysis reaction were performed, allowing calculation of time-dependent changes in bond length and the energy spacing, Δ*E*, between populated excited states and the electronic ground state. Trajectories were calculated using a velocity-Verlet algorithm with a time step of 0.2 fs and were independently propagated along one-dimensional cuts of the spin–orbit resolved 3*A*′/3*A*″/4*A*″, 5*A*″, 7*A*″/8*A*″, and 4*A*′ surfaces as calculated by Marchetti et al.^31^ and plotted in Figure 6a. The surfaces were chosen to represent a range of accessible states that lead to both I and I* products. While the dissociation process is seen to be prompt, the use of one-dimensional cuts of the potential energy surfaces assumes there is no change in the ring structure and will ignore any effects of energy redistribution into the internal degrees of freedom of the thiophenyl radical cofragment. The resulting calculations will therefore likely overestimate the velocities but will provide characteristic shifts at early times.

**Figure 6:**
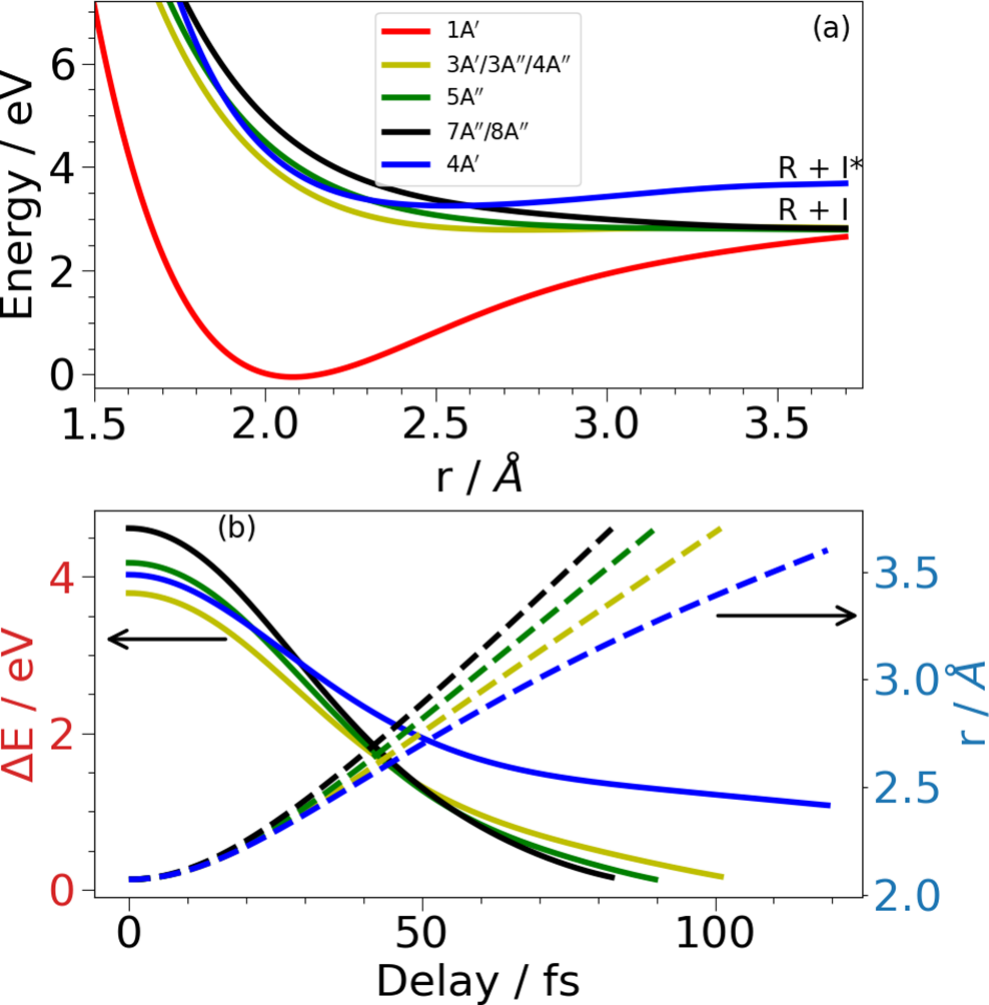
(a) Cuts along the C–I bond stretching coordinate of the spin–orbit resolved potential energy surfaces of 2-IT from Marchetti et al.^31^ used in the classical modeling of the dissociation reaction. R stands for the thiophenyl radical (C_4_H_3_S). The surfaces are reproduced from Marchetti et al.^31^ under a CC-BY license. (b) Energy spacing (solid lines, left axis), Δ*E*, and internuclear separation (dashed lines, right axis) calculated by classical dynamics simulations along the potentials in panel (a). See main text for details.

The trajectories were started from the ground state equilibrium C–I bond length of 2.07 Å,^22^ with the time-dependent changes in bond length and valence energy spacing, Δ*E*, plotted in Figure 6b. The simulations were restricted to C–I bond lengths of less than 3.75 Å, where the energy spacing between states becomes small. Over the course of the first 100 fs of the simulations, the bond extends, and the energy spacing between the excited states and the electronic ground state reduces as they approach the dissociation limit. The rate at which the changes occur depends on the shape of the potentials and on the asymptotic limit being approached. Dissociations on the one (in the current model) potential leading to spin–orbit excited iodine fragments take longer to reach 3.75 Å and maintain a larger energy gap.

To compare with the experimental measurements, the time-dependent value of Δ*E* is added to the I 4d ionization potential of the 2-IT ground state at its equilibrium geometry. This assumes that changes in the ground state ionization potential as a function of C–I bond length are minimal. While this is a simplification, previous theoretical work by Brauße et al.^1^ has shown that for the analogous system of methyl iodide, any such changes are on the order of 100 meV. In 2-IT, we can estimate the shift over the full reaction based on the changes in spectral peak position between 2-IT and atomic iodine as plotted in Figure 3a,c respectively. Based on the difference between the peak positions observed for 2-IT in the electronic ground state and the dominant peak in the atomic iodine spectrum, the shift over the entire dissociation coordinate will be on the order of 0.6 eV, significantly less than the ∼5 eV shift seen in the shake-down region.

The calculated time-dependent shake-down electron kinetic energies are overlaid on the contour map in Figure 4a as solid lines. The color coding used in Figure 4a matches that used in Figure 6 to label the different electronic states. In the interest of clarity, we only plot the expected positions for the higher kinetic energy peak associated with ionization from the I 4d_5/2_ level. The expected energies when considering both 4d features are presented in SI. The energy and shifts predicted by the model overlap well with those observed experimentally, matching the rate at which the energy shifts and highlighting that the spread in energy covered by the shake-down signal is due to dissociation pathways to the two dissociation limits.

Based on the simulations, the 78 fs delay in the appearance of the asymptotic iodine signal would correspond to a C–I bond length of 3.1–3.6 Å, depending on the potential surface populated. As the present simulations are based on one-dimensional cuts across multidimensional potential energy surfaces, these values should be taken as an upper bound on the likely C–I bond length at a 78 fs delay. These results are therefore broadly consistent with calculations by Toulson et al.^22^ which found that the X-ray absorption spectrum of the I 4d orbitals of 2-IT reached the asymptotic atomic limit (i.e., atomic iodine absorption lines would be observed) at a C–I bond length of 2.8 Å.

While a relatively simplistic model of the dynamics has been applied here, the strong dependence of the shake-down energy on geometry provides a sub-Å measure of the C–I bond length. Thus, studies of shake-down transitions provide a powerful and potentially very general tool for the investigation of motions that have large gradient differences between the populated excited state and the electronic ground state.

## Summary

In summary, the photodissociation dynamics of UV-excited 2-IT have been investigated using a time-resolved XPS probe. The TR-XPS shows characteristic peaks due to direct ionization of the excited states as well as shake-down satellite transitions that report on the energy spacing between valence excited states and the electronic ground state.

The characteristics of XPS and the shake-down process enabled a detailed analysis of the photodissociation process. Measurements at the S 2p and I 4d edges allowed conclusive identification of the excited state as being of (n/π)­σ* character. The initial shift of the shake-down signal by 4.7 eV (an energy equal to that of the pump photon) relative to the ground state signal isolates contributions associated with shake-down from the direct XPS signal. The subsequent strong dependence of the shake-down energy on the C–I bond length manifests as a 5 eV shift in the position of the shake-down transition during the photolysis reaction. Based on a simple one-dimensional model of the dynamics, the shift in the shake-down energy was mapped to a C–I bond length, thereby providing a geometry-sensitive probe of the dynamics.

The large shift seen in the shake-down signal offers a positive contrast to the direct XPS signals, where the relatively small shift of 0.6 eV is observed over the course of the photolysis reaction such that the peaks associated with the ground, electronically excited, and product states are highly overlapped, making the mapping of transient signals to transient structures impossible. The direct XPS signal could however identify the time scales of product formation with high signal contrast. This places the direct XPS measurements in line with recent X-ray absorption,^22^ X-ray scattering,^17^ and Coulomb explosion^18^ experiments performed on the same system, which measured similar time scales for product formation but could not resolve the transient structures associated with the C–I bond fission process.

With the pioneering development of seeded FEL technology at FERMI and the upcoming commissioning of seeded capabilities at FLASH, the potential for further observation, exploration, and exploitation of shake-down spectroscopy for the wider study of photochemical processes is an exciting prospect. The results present intriguing possibilities for the potential sensitivity of shake-down transitions to other excited-state processes, particularly in systems where there may be strong differences in gradients of valence state energies but not in the direct X-ray or valence photoelectron spectroscopy signals. Such differences in gradient are seen extensively in broad classes of chemical systems where UV excitation changes the bonding structure, leading to structural dynamics, and in systems where the difference in gradient leads to the formation of conical intersections where nonadiabatic transitions can occur. Future experiments will look to explore this aspect and how coupled electronic and geometric structure changes can be mapped by multisite site shake-down spectroscopy investigations.

## Supplementary Material



## Notes

Experimental details and characterization of the instrument response function; supporting measurements of the S 2p X-ray photoelectron spectrum and the pump only photoion spectrum; and extended simulations of the shake-down energies (PDF)
