# Supplementary material for: Spatial Mapping of Valence Excited-State Landscapes Using Time-Resolved Shake-Down Spectroscopy
Source: J Phys Chem A. 2026 May 22;130(22):4224–33. doi: 10.1021/acs.jpca.6c02224 (PMC13244546; doi:10.1021/acs.jpca.6c02224)
Supplement: Supplementary file 1 [file jp6c02224_si_001.pdf]

Supporting Information:

Spatial Mapping of Valence Excited-State Landscapes using Time-Resolved Shake-Down Spectroscopy

Henry J. Thompson,[†] Michele Devetta,[‡] Davide Faccialà,[‡] Rebecca A. Ingle,[¶] Stephen T. Pratt,[§] Weronika O. Razmus,[‖] Caterina Vozzi,[‡] Felix Allum,[⊥] Michael N. R. Ashfold,[△] Sonia Coriani,[∇] Raimund Feifel,[††] Ruaridh Forbes,[‡‡] David M. P. Holland,[¶¶] Daniel Rolles,[§§] Richard J. Squibb,[††] Matteo Bonanomi,[‡] Carlo Callegari,[‖‖] Marcello Coreno,[⊥⊥] Miltcho Danailov,[‖‖] Alexander Demidovich,[‖‖] Michele Di Fraia,[##] Cesare Grazioli,[⊥⊥] Michele Manfredda,[‖‖] Oksana Plekan,[‖‖] and Russell S. Minns[*,‖]

†

School of Chemistry and Chemical Engineering, University of Southampton, Highfield, Southampton, SO17 1BJ, United Kingdom

‡*CNR - Istituto di Fotonica e Nanotecnologie (IFN), 20133, Milano, Italy*

¶*Department of Chemistry, University College London, London, WC1H 0AJ, United Kingdom*

§*Chemical Sciences and Engineering Division, Argonne National Laboratory, Lemont, Illinois 60439, USA*

‖*School of Chemistry and Chemical Engineering, University of Southampton, Highfield, Southampton, SO17 1BJ, United Kingdom*

⊥*Deutsches Elektronen-Synchrotron DESY, Notkestraße85, 22607 Hamburg, Germany.*

#*Stanford PULSE Institute, SLAC National Accelerator Laboratory, 2575 Sand Hill Road, Menlo Park, CA 94025, USA*

@*Linac Coherent Light Source, SLAC National Accelerator Laboratory, 2575 Sand Hill Road, Menlo Park, CA 94025, USA*

△*School of Chemistry, University of Bristol, Bristol, BS8 1TS, United Kingdom*

∇*Department of Chemistry, Technical University of Denmark, Kgs. Lyngby, DK-2800,Denmark*

††*Department of Physics, University of Gothenburg, Gothenburg, 41296, Sweden*

‡‡*Department of Chemistry, University of California, Davis, 95616, California, USA*

¶¶*Science and Technology Facilities Council (STFC), Daresbury Laboratory, Warrington, WA4 4AD, UK*

§§*J.R. Macdonald Laboratory, Department of Physics, Kansas State University, Manhattan, 66506, Kansas, USA*

‖‖*Elettra - Sincrotrone Trieste S.C.p.A., Basovizza, 34149, Trieste, Italy*

⊥⊥*CNR-ISM, Istituto di Struttura della Materia Trieste branch, s.s. 14—km 163,5, in Basovizza Area Science Park, 34149 Trieste, Italy*

##*CNR - Istituto Officina dei Materiali (IOM), Basovizza, 34149, Trieste, Italy*

E-mail: r.s.minns@soton.ac.uk

Contents

1 Experimental Details **S2**

2 Characterisation of the instrument response function **S6**

3 Photoions **S8**

4 Simulated shake-down transition energies **S9**

5 S 2p Measurements **S9**

1 Experimental Details

The experiment was performed at the Low Density Matter (LDM) beamline at the FERMI Free Electron Laser in Trieste (Italy). Pump pulses were generated from the output of a Ti:Sapphire amplifier producing ≈800 nm pulses. Sequential second harmonic and frequency mixing processes of the ≈800 nm pulses produces the third harmonic with a wavelength of 261.9 nm and a bandwidth of 0.9 nm FWHM. The laser output was attenuated to provide 6 μJ of the 262 nm light at the interaction point at a repetition rate of 25 Hz. The UV pump was focussed to a spot size of 100 μm (HWHM) and with an estimated pulse duration of between 130 & 150 fs (FWHM). The polarisation of the pump pulses was parallel to the polarisation of the X-ray probe pulses.

Soft X-ray probe pulses were generated in the FEL-2 machine, set to produce wavelengths of 10.33 nm and 6.89 nm for the I 4d and S 2p experiments, respectively. For both FEL wavelengths, the seed laser was operated at 248 nm with the 12th harmonic generated in the first stage of the FEL. FEL-2 was then tuned to deliver either the second (10.33 nm) or third (6.89 nm) harmonic of the stage one output. Pd filters were used to suppress any residual stage one output from entering the experimental endstation. The FEL was run at

a repetition rate of 50 Hz allowing for sequential collection of pump+FEL and FEL only measurements.

A basic description of the endstation can be found in Lyamayev et al.[S1] but does not include the magnetic bottle electron spectrometer (MBES) which is described in Squibb et al.[S2]. A liquid sample of 2-Iodothiophene (Merck, 98% used without further purification), was kept in a reservoir through which 0.1 bar of helium was bubbled. The resulting vapour was subsequently expanded into vacuum via a commercial pulsed valve (Parker Series 9, 800 μm hole) operating at the repetition rate of the FEL, 50 Hz, and a nominal opening time of 110 μs. The resulting expansion was passed through a conical skimmer (Beam Dynamics model 76.2, 3 mm diameter) into the differentially pumped detection chamber before being further defined by a fixed-diameter iris (1.5 mm) and a set of piezo-operated vertical slits (Piezosystem Jena PZS 3). The molecular beam then entered the detection chamber and crossed perpendicularly to the FEL beam with the latter propagating along the horizontal short axis of the endstation. The pump-laser beam was sent into the detector chamber quasi-collinearly with the FEL (4° downward tilt).

Photoelectrons were collected in the high-resolution magnetic bottle electron spectrometer. Signals were digitised and, on a shot by shot basis, thresholded and counted to avoid artefacts associated with ringing on the signal lines. The photoelectron spectrum was calibrated by ionising the helium seed gas with 6.89 nm X-ray radiation and tracking the raw (not corrected for the TOF trigger ($\mathcal{T}_0$)) time of flight, $\mathcal{T}_{\mathrm{raw}}$, value of the He 1s^1 $^2S_{1/2}$ photoline (Binding energy of 24.587 eV[S3]) with varying retardation voltages. The time-of-flight values were converted using the following equation:

$$\begin{aligned} E_k =& \frac{1}{2} m_{\mathrm{e}} \left(\frac{L}{\mathcal{T}}\right)^2 \\ =& \left(\frac{1686.065\ L/\mathrm{m}}{\mathcal{T}/\mathrm{ns}}\right)^2 \mathrm{eV}, \end{aligned} \tag{S1}$$

where $\mathcal{T} = \mathcal{T}_{\mathrm{raw}} - \mathcal{T}_0$ is the electron time-of-flight, L is the flight length, and m_{e} is the mass

of an electron. The $\mathcal{T}_{\mathrm{raw}}$ and E_{k} data in Table S1 were fitted to equation S1 where we obtain the best-fit values $\mathcal{T}_0 = 5000 \pm 2$ ns and $L = 2.00 \pm 0.01$ m. The calibration resulting (blue) curve presented in Figure S1 was used for the S 2p data set, as no retardation was applied. Since the I 4d data set had a 47 V retardation voltage, the blue curve defines the electron kinetic energy E_k as detected as a function of $\mathcal{T}_{\mathrm{raw}}$. The orange curve defines the electron kinetic energy $E_{k,0}$ upon ionisation when retardation $V_{ret} = 47$ V was applied, as in the case for the I 4d experiment, by utilising $E_{k,0} = E_k + V_{ret}$.

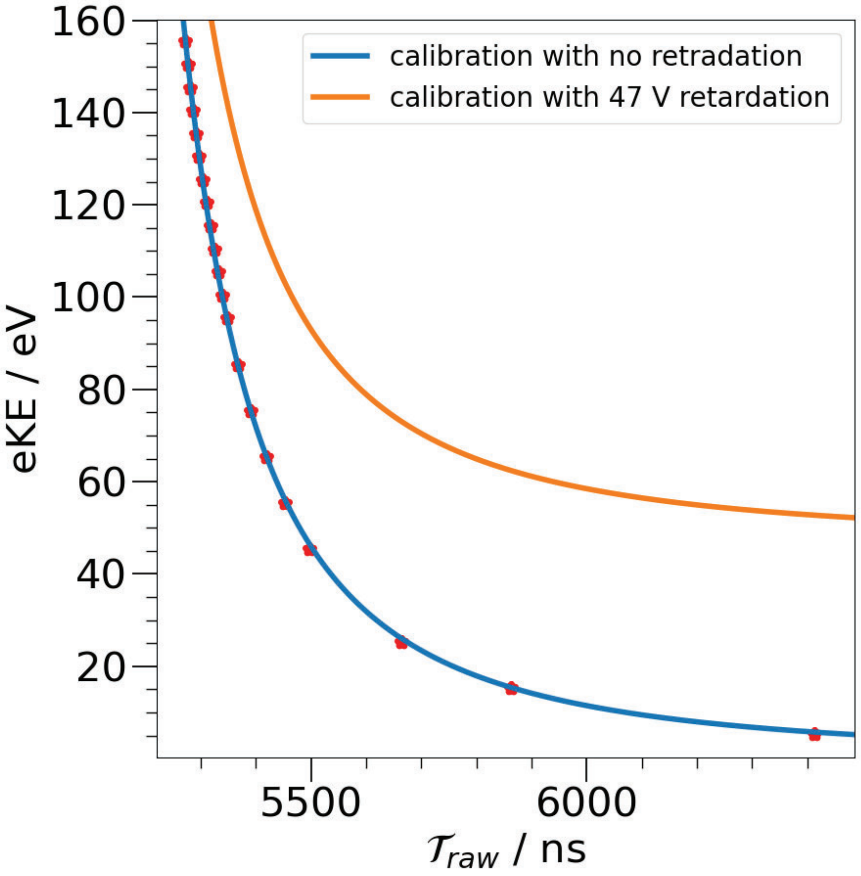

Figure S1: Time-of-flight to electron kinetic energy calibration curves resulting from the calibration data (red data points) from Table S1. The blue curve shows the measured kinetic energy at the detector, as presented in the S 2p data. The orange curve has a 47 V retardation voltage added to the kinetic energy (at the detector), allowing us to define the kinetic energy of the electron prior to the application of the retardation voltage.

Following the time-to-energy conversion, the photoelectron-time-of-flight intensity was

scaled by the Jacobian factor $|\mathrm{d}E_k/\mathrm{d}\mathcal{T}| \propto E_k^{-3/2}$ to maintain the correct area for the photoelectron-energy spectrum.

Table S1: Data used in the energy calibration of the magnetic bottle spectrometer. The He $1s^1\ ^2S_{1/2}$ photo line (eBE 24.587 eV[S3]) is formed by ionization of He $1s^2\ ^1S_0$ with a photon energy of 179.948 eV (6.89 nm) with varying retardation voltages between zero and 150 V.

$\mathcal{T}_{\mathrm{raw}}$ / ns	E_{k} / eV	V_{ret} / V
5272	155.36	0
5276	150.36	5
5281	145.36	10
5286	140.36	15
5291	135.36	20
5297	130.36	25
5303	125.36	30
5310	120.36	35
5317	115.36	40
5324	110.36	45
5331	105.36	50
5339	100.36	55
5348	95.36	60
5368	85.36	70
5391	75.36	80
5418	65.36	90
5452	55.36	100
5497	45.36	110
5663	25.36	130
5863	15.36	140
6413	5.36	150

2 Characterisation of the instrument response function

The instrument response function was characterised through measurements of sidebands created from ionisation of the helium carrier gas when the FEL and 262 nm pulses were overlapped in time. The integrated intensity of the sideband signal is plotted in Figure S2 and was fitted to a Gaussian profile that is used in the time-dependent scaled subtraction reported in the manuscript.

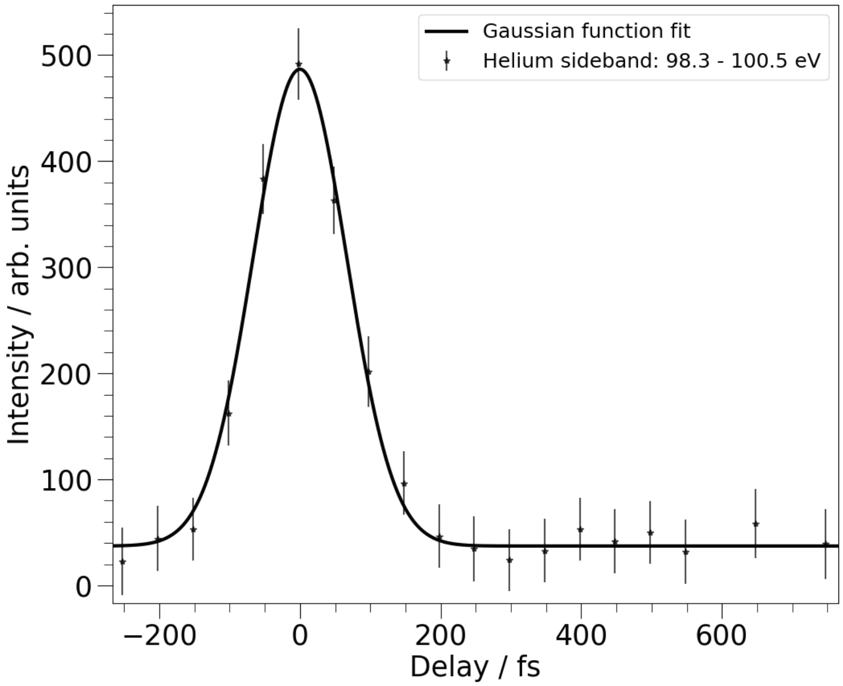

Figure S2: Integrated intensity profile of the higher kinetic energy helium sideband signal (data points) and associated Gaussian fit (solid line). The fit defines the pump-probe delay time-zero, t_0, for all presented I 4d data with an uncertainty of $\pm$ 2 fs and a cross-correlation σ value of 66 $\pm$ 2 fs (FWHM 155 $\pm$ 5 fs). Error bars on the data points represent the 2σ uncertainty obtained from a bootstrap analysis.

3 Photoions

Figure S3 shows the mass spectra of 2-iodothiophene obtained with the UV pump laser (262 nm). The pulse energy of the UV pump used to record this spectrum is 5.61 μJ, which closely matches the 6 μJ used in the TR-XPS measurements. The mass-spectrum is obtained by applying a few kV of negative electrical potential to both the magnet and the three lenses, creating a configuration that is an approximation to a two-field Wiley-McLaren mass spectrometer, allowing for collection of ions produced by the pump-pulse. The mass-spectrum highlights that at this pump power, a significant level of dissociative ionisation is occurring that contributes to the delayed rise seen in the atomic iodine signal.

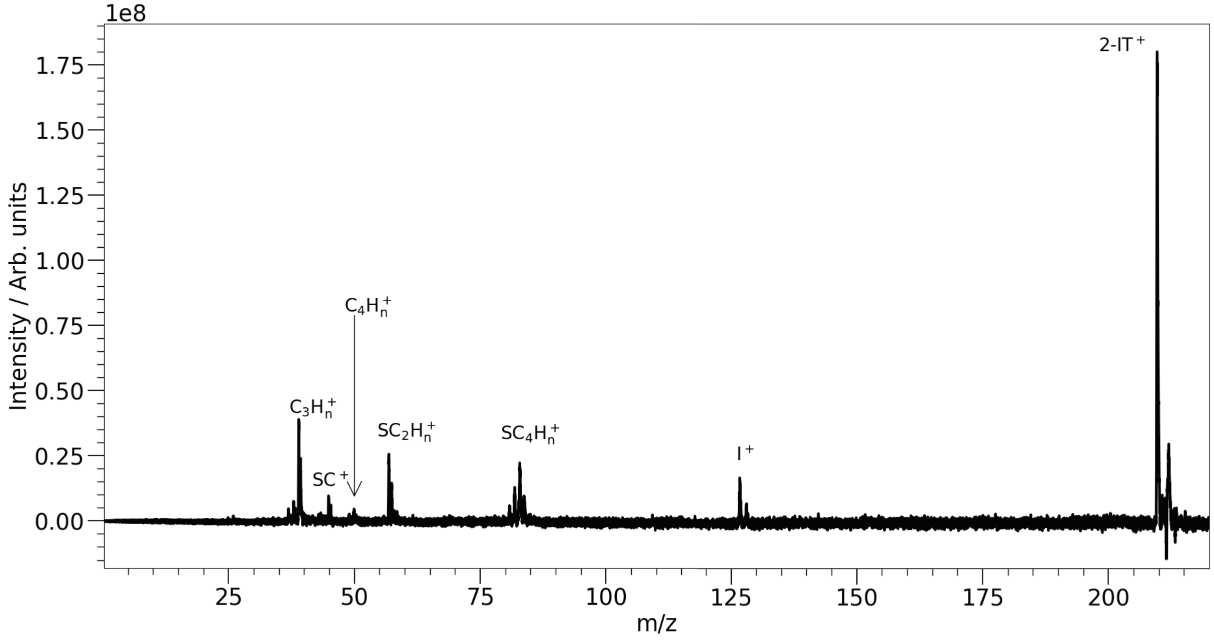

Figure S3: Mass-spectrum of 2-Iodothiophene induced by the UV pump laser (262 nm) with an average pulse energy of 5.61 μJ, and 2-iodothiophene molecules seeded in Helium.

4 Simulated shake-down transition energies

In Figure S4 we reproduce Figure 4 of the main paper but include the predicted positions of the shake-down signals associated with formation of the cation with an electron hole in the $4d_{3/2}$ levels, as well as those associated with formation of the cation with an electron hole in the $4d_{5/2}$ levels.

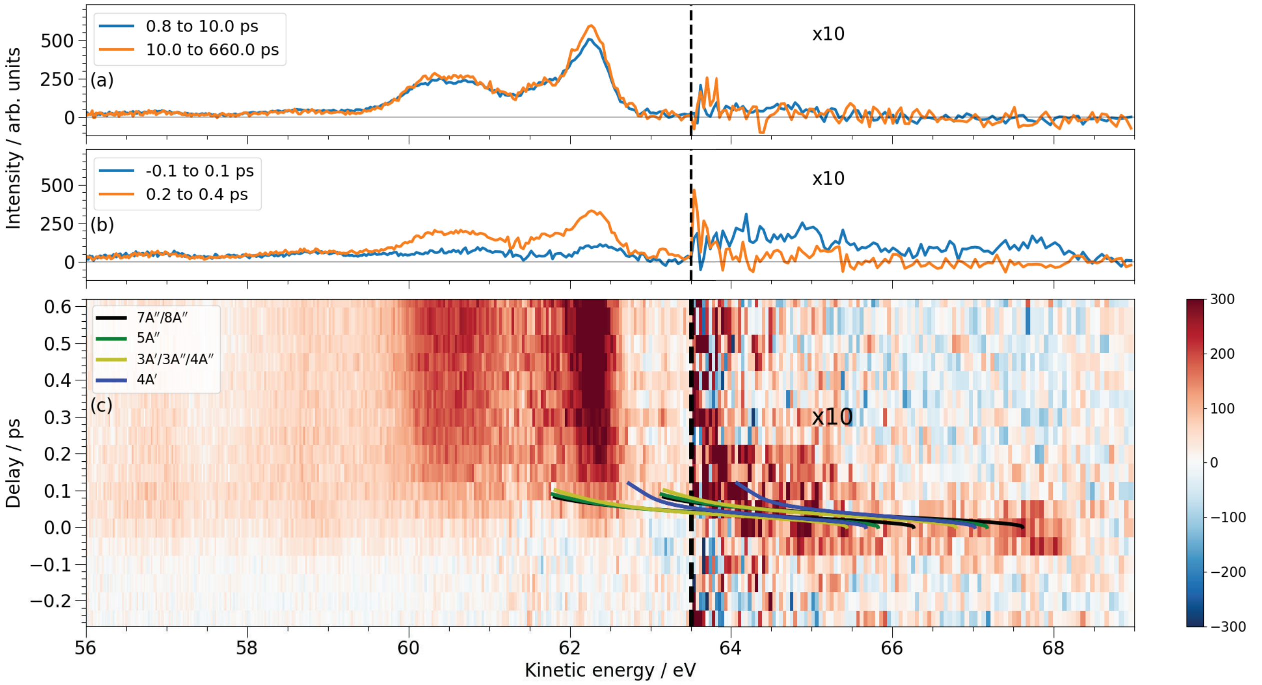

Figure S4: Time-resolved X-ray photoelectron spectrum of 2-IT (262 nm excitation and ionised with a 120 eV probe photon energy) following the time-dependent scaled background subtraction (c) described by equation **4** in the main manuscript. The solid lines overlaid on panel (c) represent the expected positions of the shake-down signals from our simulations based on the potential energy surface cuts of Marchetti et al. [S4]. The higher energy components, starting at approximately 68 eV, are identical to those plotted in Figure 4 of the main manuscript and correspond to ionisation processes that leave a hole in the $4d_{5/2}$ orbital. Lines starting from a lower initial kinetic energy, approximately 66 eV, are those associated with an ionisation processes that leave a hole in the $4d_{3/2}$ orbital. Spectra obtained at long (a) and short (b) delays provide a breakdown of the spectral features at different delay ranges.

5 S 2p Measurements

TR-XPS measurements at the S 2p edge were conducted under similar conditions to those used for I 4d. The pump laser conditions and basic endstation configuration were identical

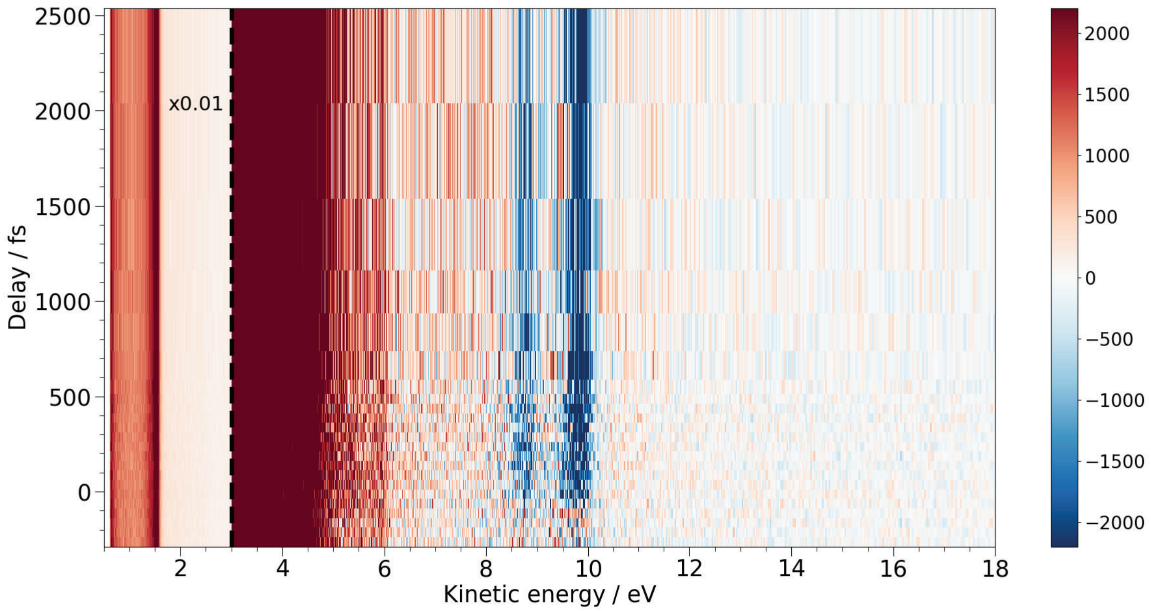

Figure S5: Time-resolved X-ray photoelectron difference (pump-on minus pump-off) spectrum of 2-IT (262 nm excitation and ionised with a 179.9 eV probe photon energy) between pump-probe delays of -265 and 2300 fs.

with the only changes being the probe X-ray photon energy of 179.9 eV and the lack of an applied retardation voltage to the magnetic bottle. The S 2p TR-XPS will therefore track the same molecular dynamics as those of the iodine spectra, but from the perspective of the sulfur atom contained within the thiophenyl ring.

The time-resolved difference (pump-on minus pump-off) colourmap is presented in Figure S5. Complicating the spectrum are signals related to multiphoton ionisation by the UV pulse that appear at kinetic energies below 6 eV. As these are single colour (pump only) signals, they show no time-dependent changes in intensity. The XPS signal associated with the neutral ground, and valence excited, states of 2-IT are expected at energies around 8-10 eV. A clear depletion of the ground state peaks of 2-IT at 8.7 and 9.9 eV, associated with formation of the S $^2P_{1/2}$ and S $^2P_{3/2}$ cation states, can be observed at time-zero but with very little change thereafter.

To monitor the timescale of the observed changes and to check for any other small changes in signal that are not immediately obvious from the colourmap, we have explored regions

of the photoelectron spectrum to see whether any time-dependent changes in intensity are present. While most regions showed no time-dependent change, the investigation highlighted changes in intensity over the energy ranges, 4.75-5.5 eV and 6.5-8.1 eV, as well as the depletion seen between 8.3-10.2 eV. These intensity changes are plotted in Figure S6.

The depletion signal observed between 8.3-10.2 eV is associated with a reduction in photoelectron intensity of the S $^2P_{1/2}$ and S $^2P_{3/2}$ signals associated with ionisation of ground state 2-IT. At time zero we also observe an enhancement of signal at 4.75-5.5 eV. The large shift to lower kinetic energies, relative to the electronic ground state, alongside the prompt appearance of this signal, leads us to attribute this increase in intensity to the population of cationic states resulting from UV multiphoton ionisation. The TR-spectra shown in Figure S5 do not show any distinct structure. This suggests that the intensity increase could be from any sulfur containing cation fragments present in the UV only mass-spectrum from Figure S3.

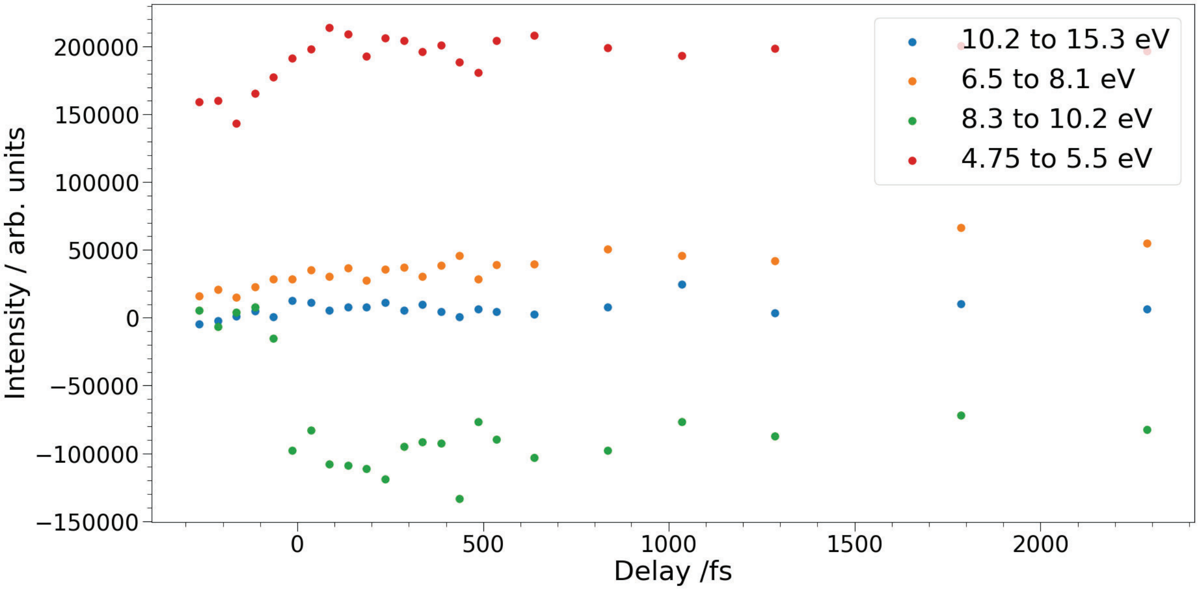

Figure S6: Summed intensity profiles over the kinetic energy ranges defined in the legend from the time-resolved S 2p difference spectra presented in Figure S5.

A small, gradual rise in photoelectron intensity is also observed between 6.5 and 8.1 eV in Figure S6. The increase in intensity is small and fairly uniform over the covered energy

range, with no discernable peaks or structure in the spectrum. While this could be indicative of a decrease in electron density around the sulfur environment from neutral (in terms of charge) dynamics, we believe this is unlikely. The absence of any structure and the long timescale over which the signal appears to grow means this does not appear correlated with the neutral dissociation process. We therefore assign this to the formation of neutral sulfur containing co-fragments generated via dissociative ionisation.

The lack of change in the S 2p spectrum attributable to neutral dissociation dynamics is perhaps surprising. As the S 2p spectrum for 2-IT has (to the best of our knowledge) not previously been reported, we use our measurements to assign ionisation energies of 171.2 and 170.0 eV to the S $^2P_{1/2}$ and S $^2P_{3/2}$ cationic states of 2-IT. To provide an estimate of the effect of the iodine atom on the positions of the peaks of these two states and to assess how the energies might shift upon dissociation we compare these to the binding energies reported for thiophene (where the iodine has been replaced with a hydrogen atom). Giertz et al.[S5] have previously reported high resolution measurements of the S 2p spectrum of thiophene and provide ionisation energies of 171.1 and 169.9 eV for the S $^2P_{1/2}$ and S $^2P_{3/2}$ ionic states respectively. The lack of any significant difference between the ionisation energies of the two molecules indicates that the presence, or not, of the iodine atom makes very little difference to the local environment of the sulfur atom and that any such shifts would be at or below the limit of our resolution in the time resolved measurement. The lack of change at early times therefore supports the assertion in the main paper that excitation into the $\pi\pi^*$ states is minimal at 262 nm and that the dominant primary step is direct excitation into the $(n/\pi)\sigma^*$ states. Fission of the C-I bond proceeds, leaving the thiophenyl radical ring structure intact with the whole process leading to minimal changes, if any, in the S2p XPS measurement over the course of the reaction.

References

(S1) Lyamayev, V.; Ovcharenko, Y.; Katzy, R.; Devetta, M.; Bruder, L.; LaForge, A.; Mudrich, M.; Person, U.; Stienkemeier, F.; Krikunova, M.; Möller, T.; Piseri, P.; Avaldi, L.; Coreno, M.; O'Keeffe, P.; Bolognesi, P.; Alagia, M.; Kivimäki, A.; Fraia, M. D.; Brauer, N. B.; Drabbels, M.; Mazza, T.; Stranges, S.; Finetti, P.; Grazioli, C.; Plekan, O.; Richter, R.; Prince, K. C.; Callegari, C. A modular end-station for atomic, molecular, and cluster science at the low density matter beamline of FERMI@Elettra. *Journal of Physics B: Atomic, Molecular and Optical Physics* **2013**, *46*, 164007.

(S2) Squibb, R. J.; Sapunar, M.; Ponzi, A.; Richter, R.; Kivimäki, A.; Plekan, O.; Finetti, P.; Sisourat, N.; Zhaunerchyk, V.; Marchenko, T.; Journel, L.; Guillemin, R.; Cucini, R.; Coreno, M.; Grazioli, C.; Di Fraia, M.; Callegari, C.; Prince, K. C.; Decleva, P.; Simon, M.; Eland, J. H. D.; Došlić, N.; Feifel, R.; Piancastelli, M. N. Acetylacetone photodynamics at a seeded free-electron laser. *Nat. Commun.* **2018**, *9*, 63.

(S3) Kandula, D. Z.; Gohle, C.; Pinkert, T. J.; Ubachs, W.; Eikema, K. S. E. Extreme Ultraviolet Frequency Comb Metrology. *Phys. Rev. Lett.* **2010**, *105*, 063001.

(S4) Marchetti, B.; Karsili, T. N. V.; Kelly, O.; Kapetanopoulos, P.; Ashfold, M. N. R. Near ultraviolet photochemistry of 2-bromo- and 2-iodothiophene: Revealing photoinduced ring opening in the gas phase? *The Journal of Chemical Physics* **2015**, *142*, 224303.

(S5) Giertz, A.; Bässler, M.; Björneholm, O.; Wang, H.; Feifel, R.; Miron, C.; Karlsson, L.; Svensson, S.; Børve, K. J.; Sæthre, L. J. High resolution C1s and S2p photoelectron spectra of thiophene. *The Journal of Chemical Physics* **2002**, *117*, 7587–7592.
